# Iron and Oxidative Stress in Parkinson’s Disease: An Observational Study of Injury Biomarkers

**DOI:** 10.1371/journal.pone.0146129

**Published:** 2016-01-11

**Authors:** Marcio S. Medeiros, Arthur Schumacher-Schuh, Andreia Machado Cardoso, Guilherme Vargas Bochi, Jucimara Baldissarelli, Aline Kegler, Daniel Santana, Carolina Maria Martins Behle Soares Chaves, Maria Rosa Chitolina Schetinger, Rafael Noal Moresco, Carlos R. M. Rieder, Michele Rechia Fighera

**Affiliations:** 1 Neurology Service, Hospital de Clínicas de Porto Alegre, Porto Alegre, Rio Grande do Sul, Brazil; 2 Natural and Exact Sciences Center, Graduate Program in Life Sciences: Toxicological Biochemistry, Universidade Federal de Santa Maria, Santa Maria, RS, Brazil; 3 Department of Clinical and Toxicological Analyses, Universidade Federal de Santa Maria, Health Sciences Center, Santa Maria, Rio Grande do Sul, Brazil; 4 Neuropsychiatry Department, University Hospital, Universidade Federal de Santa Maria, Health Sciences Center, Santa Maria, Rio Grande do Sul, Brazil; The Pennsylvania State University Hershey Medical Center, UNITED STATES

## Abstract

Parkinson's disease (PD) is characterized by progressive motor impairment attributed to progressive loss of dopaminergic neurons in the substantia nigra (SN) pars compacta. In addition to an accumulation of iron, there is also an increased production of reactive oxygen/nitrogen species (ROS/RNS) and inflammatory markers. These observations suggest that iron dyshomeostasis may be playing a key role in neurodegeneration. However, the mechanisms underlying this metal-associated oxidative stress and neuronal damage have not been fully elucidated. To determine peripheral levels of iron, ferritin, and transferrin in PD patients and its possible relation with oxidative/nitrosative parameters, whilst attempting to identify a profile of peripheral biomarkers in this neurological condition. Forty PD patients and 46 controls were recruited to compare serum levels of iron, ferritin, transferrin, oxidative stress markers (superoxide dismutase (SOD), catalase (CAT), nitrosative stress marker (NOx), thiobarbituric acid reactive substances (TBARS), non-protein thiols (NPSH), advanced oxidation protein products (AOPP), ferric reducing ability of plasma (FRAP) and vitamin C) as well as inflammatory markers (NTPDases, ecto-5’-nucleotidase, adenosine deaminase (ADA), ischemic-modified albumin (IMA) and myeloperoxidase). Iron levels were lower in PD patients, whereas there was no difference in ferritin and transferrin. Oxidative stress (TBARS and AOPP) and inflammatory markers (NTPDases, IMA, and myeloperoxidase) were significantly higher in PD, while antioxidants FRAP, vitamin C, and non-protein thiols were significantly lower in PD. The enzymes SOD, CAT, and ecto-5’-nucleotidase were not different among the groups, although NOx and ADA levels were significantly higher in the controls. Our data corroborate the idea that ROS/RNS production and neuroinflammation may dysregulate iron homeostasis and collaborate to reduce the periphery levels of this ion, contributing to alterations observed in the pathophysiology of PD.

## Introduction

Oxidative stress is a present factor throughout the average aging process. However, it is higher in at least 60 different age-related diseases, such as Parkinson’s disease (PD) [1]. In PD, oxidative stress is a result of mitochondrial deficiency, in addition to a chronic inflammatory process, in which both produce reactive oxygen species (ROS) and reactive nitrogen species (RNS). These reactive species meet the accumulated iron in the brain and harm structures, leading to the death of dopaminergic neurons in the substantia nigra. This process creates a cycle of cell damage, neuroinflammation, and ROS/RNS production, resulting in neuronal death [2].

Previous studies suggest that altered iron homeostasis may be involved with PD pathogenesis, where lower levels of serum iron were found in individuals who developed the disease [3, 4]. Furthermore, the results in peripheral levels of superoxide dismutase (SOD), catalase (CAT), and glutathione peroxidase (GPx) are controversial and unreliable to measure oxidative stress in PD [5]. Other oxidative stress and inflammatory markers are also being studied: advanced oxidation protein products (AOPP), ferric reducing ability of plasma (FRAP), nitric oxide (NO), thiobarbituric acid reactive substances (TBARS), vitamin C and non-protein thiols (NPSH) as well as ATP and ADP NTPDases, ecto-5’-nucleotidase, adenosine deaminase (ADA), myeloperoxidase and ischemic-modified albumin (IMA).

Some of these different markers have not yet been tested in PD and may be useful in the study of this pathogenesis. Thus, understanding iron homeostasis and its relation with oxidative/nitrosative stress could find reliable parameters that may be related to the neurodegenerative process in PD.

## Methods

This study included 40 patients with PD. The diagnosis was in accordance with the UK Parkinson’s Disease Society Brain Bank Research criteria. The diagnostic criteria were revised by a neurologist with experience in movement disorders [6]. The PD patients were recruited from Hospital de Clínicas de Porto Alegre and invited to volunteer for the study. Atypical and secondary parkinsonisms were excluded from the study. In addition to patients with chronic renal, inflammatory, liver, and hematological diseases and cancer history, active cigarette smoking was also an exclusion criterion.

For the control group, 46 healthy subjects were recruited from a group of volunteers in the city of Santa Maria. The mean age and sex were similar among the groups. The same exclusion criteria used with the PD patients was applied. Sociodemographic information was collected and a physical examination including weight and height to calculate body mass index (BMI) was performed. All PD patients and controls answered a mini-nutritional questionnaire in order to exclude a possible deficit in nutrient ingestion. Blood samples were taken from all PD patients and healthy controls. For the PD patients, the Unified Parkinson’s Disease Rating Scale (UPDRS) and Hoehn & Yahr (H&Y) were also performed.

Biochemical analysis were performed in laboratories at Universidade Federal de Santa Maria. These analyses included the measurement of iron metabolism in serum sample (iron, ferritin, and transferrin), oxidative stress markers (SOD, CAT, TBARS, AOPP), nitrosative stress (NOx), inflammatory response markers (NTPDase ATP, NTPDase ADP, ecto-5’-nucleotidase, ADA, myeloperoxidase, IMA), and antioxidant activity (FRAP, vitamin C, NPSH).

The study was approved by the local Ethical Committee and carried out in accordance with The Code of Ethics of the World Medical Association (Declaration of Helsinki). All subjects participated after providing written informed consent.

### Mini Nutritional Avaliation

The Mini Nutritional Avaliation (MNA) covers 18 questions related to anthropometric evaluation (weight, height, and weight loss); general evaluation (lifestyle, medicament use, mobility); dietetics evaluation (number of meals, food intake, autonomy to eat alone) and self-evaluation (health perception and nutritional status). The first 6 questions comprehend a nutritional screening. Each answer received a value, which was then added to make a final score. Elderly people that present at least 12 points have an interrupted evaluation. The next 12 questions comprehend a global evaluation. A total score (screening and global evaluation) higher or equal to 24 indicates a good nutritional status; from 17 to 23.5 indicates a malnutrition risk and lower than 17, malnutrition [7].

### Anthropometric Evaluation

Body weight was assessed by a weighing scale, whereas body height measurement was done with the altimeter from the weighing scale. Arm circumference (AC) and calf circumference (CC) were measured with a non-elastic tape measure. AC and CC measurements were assessed to complete the MNA and were evaluated separately. BMI was measured in accordance with the formula: BMI = actual weight (kg)/(height)² and classified according to Lipschitz parameters [8].

### Biochemical analysis

Blood samples were collected after an overnight fast by venous puncture technique into Vacutainer^®^ (BD Diagnostics, Plymouth, UK) tubes with no anticoagulant. AOPP was assessed as previously described by Hanasand *et al*. [9]. For IMA, the technique described by Kaefer *et al*. [10] was used. NOx was estimated according to Tatsch *et al*. [11], and FRAP was measured as described by Benzie *et al*. [12].

Lipid peroxidation was estimated by measuring TBARS in serum samples according to a modified method of Jentzsch *et al*. [13]. NPSH were assayed in plasma and erythrocytes by the method of Ellman [14].

The determination of CAT activity was carried out in accordance with a modified method of Nelson & Kiesow [15]. SOD activity was measured as described by McCord & Fridovich [16]. Vitamin C analysis was determined by the modified method described by Jacques-Silva *et al*. [17].

Protein was measured by the Bradford method [18] using bovine serum albumin as standard. Platelet-Rich Plasma (PRP) was prepared by the method of Lunkes *et al*. [19].

NTPDase measurement and ecto-5′-nucleotidase activity were carried out as described by Lunkes *et al*. [19]. ADA activity determination was performed as described by Giusti and Gakis [20].

Myeloperoxidase activity was measured in plasma from blood collected with EDTA and followed by centrifugation at 1800 x g for 10 min. The activity was analyzed spectrophotometrically by a modified peroxidase-coupled assay system involving phenol, 4-aminoantipyrine (AAP) and H_2_O_2_ [21]. Serum was routinely centrifuged at 2500 x g for 15 min and used to assess the levels of iron, transferrin, and ferritin. The analysis of iron, transferrin, and ferritin were performed using standard methods on cobas mira (Roche Diagnostics, Basel, Switzerland).

### Statistical analysis

The Shapiro-Wilk test was performed to verify if the variable had normal distribution. Statistical analysis were done comparing the results from PD patients and controls using Student t test for normal variables, Mann-Whitney for non-parametric data or Chi-square test when appropriate.

## Results

A total of 40 patients with PD were included from January 2012 to December 2012. The control group consisted of 46 healthy individuals from a group of volunteers to match age and sex. Among the cases, 45% were men and 55% were women; the average age of cases was 65.95 years; mean BMI was 25.82; mean disease duration was 8.57 years. The distribution by age, sex, and BMI was similar in controls. Individuals in the PD group had mini-nutritional forms with normal scores (with index ≥ 24). Table 1 shows the sociodemographic data of the case-control analysis.

**Table 1:** Sociodemographic information of patients with Parkinson’s disease and controls.

	Parkinson’s disease (N = 40)	Control (N = 46)	
	Mean (±SD) or N(%)	Mean (±SD)	P
Age (years)	65.95 (±12.3)	62.30 (±10.17)	0.136^a^
Male	18 (45%)	19 (41%)	0.73^b^
BMI	24.44 (±4.19)	25.82 (±3.98)	0.12^a^
Mini-nutritional questionnaire	24.42 (± 5.60)	26.86±2.19	0.250^a^
Disease duration (years)	8.57 (±5.91)		
UDPRS	44.26 (±18.96)		
H&Y			
1	0 (0)		
2	21 (52.5%)		
3	18 (45.0%)		
4	1 (2.5%)		
5	0 (0)		

Data are presented as median ± interquartile range or means ± SD for n = 40–46 in each group. No significant differences between groups were detected. For PD patients, the Unified Parkinson’s Disease Rating Scale (UPDRS) and Hoehn & Yahr (H&Y) were utilized. Statistical analyses employed were

^a^Student’s t test and

^b^Chi-square test.

### Iron level

Data on iron, ferritin, and transferrin levels were collected from all 40 cases and 46 controls (Table 2). Iron levels were lower between cases (67.5 μg/dL) compared to controls (78 μg/dL, P = 0.034). On the other hand, ferritin levels in PD patients (142.5 ng/mL) were not significantly different when compared to controls (161 ng/mL; P = 0.499). Transferrin levels in PD patients (273.20 mg/L) were also not significantly different when compared to controls (253.80mg/L; P = 0.065).

**Table 2:** Comparison of iron, ferritin, and transferrin peripheral blood levels between patients with Parkinson’s disease and controls.

	Parkinson’s disease	Control	P
Iron (μg/dL) (median-IR)	67.50 (56.5–82)	78 (64.5–89)	0.034^a^*
Ferritin (ng/mL) (median- IR)	142.5 (73.7–255)	161 (101.5–221)	0.499^a^
Transferrin (mg/L) Mean (±SD)	273.20 (±49.1)	253.80 (±44.8)	0.065^b^

Data are presented as median ± interquartile range and means ± SD for n = 40–46 in each group. Statistical analyses employed were ^a^Mann-Whitney U test and ^b^Student’s t test.

*P was significant when P<0.05.

Iron levels were lower between cases compared to control. Ferritin and transferrin levels were not significantly different between groups.

### Biomarkers

Although not all individuals were analyzed and some samples were lost en route to the laboratory due to transportation difficulties, analyses concerning oxidative stress markers presented significant differences comparing cases and controls. All results are described in Table 3. Biomarkers of oxidative damage were higher in the PD group. Among the antioxidants, the tendency was to find decreased levels in PD patients in comparison to controls. The oxidative stress markers SOD (P = 0.221) and CAT (P = 0.403) were not significantly different between the groups. NOx was used as an estimation of NO. Statistical analysis revealed that the control group presented higher NO levels (196.5 μmol/L; P = 0.004) than the PD group (112.8 μmol/L).

**Table 3:** Comparison of oxidative stress and antioxidant peripheral biomarkers between patients with Parkinson’s disease and control.

Oxidative/ nitrosative markers	N (DP/Control)^a^	Parkinson´s disease Median (interquartile range)	Control Median (interquartile range)	P
IMA (Absorbance)	40/46	0.58 (0.51–0.68)	0.55 (0.46–0.59)	0.008*
AOPP (μmol/L)	40/46	65.6 (52.43–81.38)	45.6 (37.58–62.78)	<0.001*
NOx (μmol/L)	40/46	112.8 (77.55–160.93)	196.5 (99.9–256.0)	0.004*
SOD (U/mg hemoglobin)	40/46	6.25 (4.24–8.34)	6.63 (4.85–8.61)	0.221
Catalase (U/g hemoglobin)	40/46	9.9 (8.73–13.60)	11.5 (8.8–13.9)	0.403
TBARS (nmol MDA/mL serum)	40/46	11.72 (9.48–14.29)	8.89 (7.83–10.58)	0.001*
NTPDase (ATP) (nmolPi/min/ mg/protein)	34/41	20.10 (13.58–25.18)	15.07 (9.80–18.97)	0.013*^a^
NTPDase (ADP) (nmolPi/min/ mg/protein)	34/42	23.79 (18.33–33.73)	14.69 (10.11–18.75)	< 0.001*^a^
Ecto-5´nucleotidase (nmolPi/min/ mg/protein)	35/41	19.01 (8.69–25.52)	13.76 (9.68–19.36)	0.1^a^
Myeloperoxidase	40/46	2.14 (1.28–3.05)	1.31 (1.07–2.50)	0.02*
ADA (UI/L)	35/37	1.61 (1.17–3.22)	2.81 (1.57–7.47)	0.049*^a^
**Antioxidant markers**				
NPSH (nmol NPSH/mL of platelet)	40/46	0.91 (0.60–0.95)	0.97 (0.94–1.04)	< 0.001*
FRAP (μmol/L)	40/46	587.5 (516.5–696.8)	895.5 (825.3–989.3)	<0.001*
Vitamin C (μmol/L serum)	40/46	17.81 (8.70–35.52)	34.92 (24.75–52.43)	<0.001*

Data are presented as median ± interquartile range for n = 40–46 in each group. Statistical analysis employed was Mann-Whitney *U* test.

*P was significant when P<0.05,

^a^data lost in the preparation process.

No correlations of iron or any of the oxidative and antioxidative markers with disease duration were found in the UPDRS and the H&Y scores (data not shown).

## Discussion

Significant results concerning iron levels and different oxidative/nitrosative and inflammatory biomarkers were obtained in the present study. Some of these biomarkers have not yet been tested in PD: AOPP, FRAP, NTPDase, IMA, and myeloperoxidase.

Moreover, we observed a significant difference in the blood concentration of iron when comparing PD patients to healthy subjects. Conversely, ferritin and transferrin levels were not different between the groups.

Controversial results regarding iron blood levels in PD patients have been found [3, 4, 22]. Notably, Berg *et al*. [23] demonstrated a negative correlation between substantia nigra (SN) echogenicity, which reflects high iron content and serum levels of iron in PD patients. A case-control study suggests that an increase in the risk of PD in men with a history of multiple blood donations is due to having a low level of iron storage [24]. Pichler *et al*. [4] demonstrated that a protective effect was found for PD of higher iron levels with a 3% (p = 0.001) reduction of relative risk for every increase of 10 mg/dL of serum iron.

The lower levels of peripheral iron in these patients may reflect a disturbance in the control of iron homeostasis [25] and a possible restriction of iron intake during life [3]. Although iron accumulates in the SN and is therefore considered a risk factor for the development of PD, a low level in peripheral blood is also associated with an increased risk. This occurs possibly because it reduces the functioning of neuronal enzymes, since it is a cofactor of tyrosine hydroxylase and has a role in the synthesis of neurotransmitters. Furthermore, low peripheral iron may decrease ferritin storage in neurons, thus decreasing the pool of iron available for neuronal enzymes and consequently leading to accumulation of free iron in the SN [4].

No significant difference was observed in ferritin and transferrin levels between PD patients and the control group in our study. However, although the ferritin mechanism in the neurodegeneration is unclear, several studies show its relationship with some neurodegenerative disorders. Devos *et al*. [26] observed that CSF ferritin is elevated in PD, and a decrease of CSF ferritin upon treatment with an iron chelator coincided with improved symptoms. Previous studies have already mentioned that dysregulation of iron metabolism may be important for Parkinson’s disease progression [27, 28]. In addition, elevated CSF ferritin has also recently been shown to predict outcomes of Alzheimer’s disease [29]. Thus, future studies are required to clarify the role of ferritin in Parkinson’s disease and other neurodegenerative diseases.

Since NO has been linked to iron metabolism [30] and proposed as a major upstream event in PD pathogenesis [31], we evaluated the involvement of nitrosative stress in this disease. In our study, it was observed a significant decrease in NOx levels in the PD group when compared to controls. In fact, Tuncel *et al*. [32] also presented reduced NO levels in patients with PD. A likely cause for these low levels may be a defective NO-dependent adaptation mechanism or the exhaustion of NO storage in the course of PD [33, 34, 35]. Another hypothesis is that NO may induce iron deprivation by reducing ion export from substance nigra (SN) to periphery [36], which could also explain why NO and iron serum levels are both decreased. Thus, low peripheral iron probably reflects altered iron trafficking (leading to both iron accumulation in SN and low peripheral iron) mediated by upstream NO levels alteration, contributing to the symptoms observed in PD. However, other studies should be realized to clary this point.

Notably, NO levels in PD are ambiguous and studies on nitrite and nitrate measurements in the cerebrospinal fluid (CSF) of PD patients have been contradictory, as there was no change [37], increase [38] or decrease [39, 40]. On the other hand, some studies have shown that CSF and plasma nitrate levels did not correlate with age at onset, duration, scores of the unified Parkinson’s disease rating scales, and Hoehn and Yahr staging in the patients group [37, 41]. Likewise, NO-mediated neurotoxic and neuroprotective effects seem to be dependent of its oxidative/reductive status, and the exact contribution to neurodegeneration is still not completely understood.

Indeed, there are sensitive and specific assays to measure NO, including performance liquid chromatography (HPLC) and Gas chromatography-mass spectrometry (GC-MS). Nevertheless, the Griess method is recommended for nitrate and nitrite quantification because it is easier, quicker, cheaper, and requires a shorter amount of time than HPLC and GC-MS [42].

The antioxidants vitamin C, NPHS and FRAP were all significantly lower in the PD group when compared to controls. Several previous studies demonstrate a decreased antioxidant activity in different diseases, even neurodegenerative disorders such as ALS [43, 44, 45]. Vitamin C and glutathione are non-enzymatic antioxidants, which have been associated with PD previously [43, 46, 47]. Our data corroborates with this idea, meaning that patients have either insufficient or defective vitamin C levels. Furthermore, pathologic data confirms lower glutathione levels in the SN of PD patients than in the control group [48].

FRAP estimates total antioxidant activity of plasma, which contains vitamin C, vitamin E, bilirubin, and uric acid [49], although it has been described in different neurological diseases presenting a low level of antioxidant activity [50]. To our knowledge, this is the first report of FRAP in PD. The low levels of serum antioxidants indicate a deficiency in this mechanism, creating an imbalanced environment where oxidants prevail and damage cellular structures. Whether this decreased antioxidant activity is primary or secondary in the neurodegenerative process remains to be elucidated.

To evaluate defense antioxidant enzymatic, we also determined SOD and CAT activity of PD and control group. No significant difference was noted in activity when comparing groups. Although no different results were found in our study, previous research has shown that SOD and CAT activity present high as well as low levels in PD and control group [5, 36]. These enzymes have been used as peripheral biomarkers of oxidative stress due to enzymatic antioxidants involved in the degradation of H_2_O_2_ into water [51]. In this way, considering that SOD and CAT are reliable parameters for oxidative stress, their lower activity although not significant, may suggest an improper functioning of cellular antioxidant defense.

Indeed, lipid damage (TBARS) and protein (AOPP) levels were significantly elevated in the PD group. TBARS is used as a parameter of oxidative stress in PD due to its identification in the SN of these patients by Dexter *et al*. [52]. This technique is associated with the degradation of lipids that participate in the lipid peroxidation process. The process begins with initial damage to a lipid by either oxidative or nitrosative stress [53], thus creating a lipid radical that continues to harm different structures until it becomes stable again and ends the oxidative stress cycle. In fact, recent studies have confirmed damage to α-synuclein by lipids in PD individuals [54]. Additionally, TBARS also indicates oxidative stress in other neurodegenerative disorders: Alzheimer’s disease and amyotrophic lateral sclerosis (ALS) [51, 54].

AOPP has been used to measure damage done to proteins by oxidative stress in different neurological pathologies, such as multiple sclerosis, amyotrophic lateral sclerosis, and mitochondrial myopathies [50]; however, there are none describing its elevation in PD. To our knowledge, this is apparently the first report of AOPP in PD. Thus, the data presented in this study suggests TBARS as a reliable parameter of oxidative stress in PD and introduces AOPP as an interesting marker that measures protein damage linked to oxidative stress in PD.

On top of that, the data presents oxidative stress parameters associated with significantly increased inflammation in PD patients. The NTPDase ATP/ADP activities were higher in PD patients, suggesting an important inflammatory activity in this disease. These enzymes degrade ATP and ADP, which are elevated in pro-inflammatory conditions where they stimulate lymphocytes and cytokines. Previous studies have shown its elevation in systemic diseases and in multiple sclerosis [55, 56, 57]; however there are no data in neurodegenerative disorders despite inflammation being a known process in these diseases. Although no significant difference was obtained, ecto-5’-nucleotidase activity was higher in PD patients, and this may occur due to losses in the samples used to measure it. This enzyme follows the degradation of ADP and acts on AMP.

Myeloperoxidase activity was significantly higher in PD patients. In addition to this enzyme being present in inflammatory conditions and its importance in the battle against infectious agents, it may also react with ROS and form strong free radicals such as hydroxyl radical and peroxynitrite [58, 59]. Moreover, despite the fact that it has been described in cardiovascular diseases [58], there are no data in neurodegenerative disorders.

IMA had significantly elevated levels in PD patients. It was first described in cardiac ischemic events, modifying the N-terminal region of albumin. Later studies found increased levels of IMA in rheumatologic, autoimmune, and neoplastic diseases [60], thus confirming its inflammatory effect in different conditions. However, no studies were conducted in neurodegenerative disorders. Although IMA levels are dependent of albumin, they were not measured in this study. Nonetheless, no difference in albumin between the studied groups may be presumed, since the patients had normal mini-nutritional scores and BMI did not differ between groups.

ADA was the exception in the biomarkers group since it had significantly higher levels in the control group. This enzyme has already been studied in diseases with pro-inflammatory states such as cancer and was elevated in the disease group [61]. ADA decreases adenosine levels, a known anti-inflammatory agent and could be a biomarker for neurodegeneration. The possibility of individuals with unknown cancer in the control group cannot be excluded and may explain this finding.

Thus, we could suggest that TBARS, AOPP, myeloperoxidase and NTPDase ATP/ADP activities, Vitamin C, glutathione, and FRAP may be reliable parameters to be used in future studies in PD.

There are no laboratory tests utilizing blood, cerebrospinal fluid, or urine samples that have proven to be effective in the primary diagnosis confirmation of PD [62]. Many potential protein biomarkers in the blood and cerebrospinal fluid have been pursued for the diagnosis and staging of PD. DJ-1 and α-synuclein, two proteins critically involved in PD pathogenesis, have tested as potential disease biomarkers, although results have been inconsistent [63, 64]. An elegant study of Han *et al*. [65] showed that some autoantibody biomarkers (for example: ICAM4, Myotilin, Fibronectin 1, Pentatricopeptide repeat domain 2) can distinguish PD and health controls, multiple sclerosis, Alzheimer disease and cancer with 90–100% accuracy. However, additional studies are required in order to further understand the role of biomarkers in Parkinson’s disease.

There are some limitations in this study that should be addressed. Firstly, we did not evaluate what the patients and health subjects ate the night before blood collection. Nevertheless, PD patients and control answered the mini-nutritional evaluation (MNA) prior to blood collection. In addition, even though serum albumin was not measured, this measurement is important to control IMA levels since it depends on plasmatic albumin levels. However, according to the mini-nutritional questionnaire, neither PD nor control subjects presented nutritional deficiency. Furthermore, Paré *et al*. [66] revealed that PD patients are not associated with significant albumin alterations in blood samples. The possibility of different diseases such as cancer and other inflammatory conditions that can alter the levels of oxidative/nitrosative markers cannot be excluded in both groups. Future studies with larger samples, basic screening for neoplastic diseases, albumin levels, and a food intake log prior to blood collection should be considered in order to confirm the data found in this research and minimize confounding factors.

## Conclusion

Iron is considered a risk factor for PD because it accumulates in the SN of patients. However, we found decreased iron concentrations in the peripheral blood, indicating that this accumulation is not dependent of an iron overload, but probably due to iron homeostasis dysregulation.

Taken together, the data presented in this study supports the role of oxidative stress in the pathophysiology of PD and suggests that FRAP, vitamin C and glutathione can be useful parameters of antioxidant activity in PD. Inflammation is also a part of the disease process and our data presents myeloperoxidase, NTPDase, IMA and possibly ecto-5’-nucleotidase, which seem to be reliable parameters of inflammatory activity in PD.

Thus, our results corroborate the idea of low levels of antioxidants, incapable of controlling free radical and ROS/RNS production with subsequent inflammation, leading to neurodegeneration in PD.
